# Predictors of patient self-report of chronic kidney disease: baseline analysis of a randomised controlled trial

**DOI:** 10.1186/s12875-014-0196-3

**Published:** 2014-11-30

**Authors:** Hannah Gaffney, Thomas Blakeman, Christian Blickem, Anne Kennedy, David Reeves, Shoba Dawson, Rahena Mossabir, Peter Bower, Caroline Gardner, Victoria Lee, Anne Rogers

**Affiliations:** NIHR Collaboration for Leadership in Applied Health Research (CLAHRC) Greater Manchester, Centre for Primary Care, Institute of Population Health, University of Manchester, Oxford Road, Manchester, M13 9PL UK; NIHR CLAHRC Wessex, Health Sciences, University of Southampton, Highfield Campus, 12 University Road, Southampton, SO17 1BJ UK; NIHR School for Primary Care Research, Centre for Primary Care, Institute of Population Health, University of Manchester, Oxford Road, Manchester, M13 9PL UK

**Keywords:** Kidney diseases, Primary health care, Self-report, Awareness, Predictors, Communication, Self-management

## Abstract

**Background:**

Improving the quality of care for patients with vascular disease is a priority. Clinical guidance has emphasised the importance of early identification and active management of chronic kidney disease (CKD) in primary care in order to maintain vascular health. However, awareness of stage 3 CKD amongst patients remains limited. We aimed to identify predictors of patient self-report of CKD to inform tailoring of conversations around CKD in primary care for diverse patient populations.

**Methods:**

We conducted a cross-sectional analysis of baseline data from 436 patients with stage 3 CKD from 24 GP practices taking part in a randomised controlled trial (RCT) evaluating a complex self-management intervention, which aimed to support the maintenance of vascular health in patients with stage 3 CKD. Potential predictors of patient self-report of CKD included demographics, stage of CKD, cardiovascular risk, self-reported co-morbidities, health status, self-management ability, and health service utilisation.

**Results:**

Around half (52%, n = 227) of patients did not self-report CKD. Self-report rates did not appreciably differ by practice. Multivariate analysis revealed that female patients (*p* = 0.004), and patients with stage 3b CKD (*p* < 0.001), and with higher anxiety levels (*p* < 0.001), were more likely to self-report CKD.

**Conclusions:**

Self-report of kidney problems by patients on CKD registers was variable and patterned by sociodemographic factors. Although it cannot be assumed that failure to self-report indicates a lack of awareness of CKD, our data do suggest the need for greater consistency in discussions around kidney health, with meaningful and relevant clinical dialogue that is aligned with existing clinical encounters to enable shared decision making and minimise anxiety.

**Electronic supplementary material:**

The online version of this article (doi:10.1186/s12875-014-0196-3) contains supplementary material, which is available to authorized users.

## Background

Chronic Kidney disease (CKD) is a growing public health concern. Clinical guidelines emphasise the importance of early identification and active management of CKD in maintaining vascular health in primary care [1]. This reflects evidence that CKD is an independent cardiovascular risk factor and that individuals with stable CKD are approximately 20 times more likely to die from cardiovascular disease than progress to end stage renal failure [2,3]. Recognising this risk, CKD has been incorporated into more recent cardiovascular risk calculators [4] and the introduction in 2006 of renal domains within the Quality and Outcomes Framework (QOF) (a UK pay-for-performance contract designed to improve quality of care) encourages regular monitoring of blood pressure for patients with stages 3 to 5 CKD (further detail in methods section below) [5]. Based upon best available evidence, indicators of quality have been assigned to a range of long-term conditions. General practices are then paid for delivering care in line with these QOF quality indicators. Nevertheless, there is evidence that the prevalence could be greater than that apparent in QOF CKD registers suggesting under-recognition in primary care [6].

The UK based national guidance NICE (National Institute for Health and Care Excellence) Quality Standards state that people with CKD are assessed for cardiovascular risk, and guidance highlights a need to offer information, education and lifestyle advice [1,3]. However, awareness of CKD among patients and practitioners is variable [7] and according to a recent UK based study, 41% of patients with stage 3 CKD were unaware of a CKD diagnosis as defined by self-report [8]. Moreover, the frequency of clinical discussion about CKD is considerably lower (26%) compared to discussions related to diabetes (60%), hypertension (72%) and medication adherence (89%) [9]. Improving the knowledge and personal skills of individuals with long-term conditions through shared decision-making is central to provision of self-management support in the UK [10-13]. A basic prerequisite for shared decision making and effective self-management education and support is awareness of a diagnosis [14].

Explanations for variation in the self-reporting of CKD may include considering reduced kidney function amongst older patients as a natural part of the ageing process [15,16]. Primary care professionals may be cautious not to over-diagnose CKD and have expressed concerns about the disclosure of CKD to patients [17,18], particularly around raising unnecessary anxiety in the elderly where clinical benefit was felt to be less certain [19]. Previous studies suggest that patients who are male and of younger age [8,16], who have CKD stage 3b with proteinuria [8,16,19], additional vascular risk factors [7,16] or established vascular disease, are more likely to self-report CKD.

The primary aim of this study is to assess what factors (demographic, clinical and psychological) are associated with self-report of CKD. This will inform how information exchange around CKD in primary care could be tailored to diverse patient populations. It builds on work conducted within the NIHR CLAHRC (Collaboration for Leadership in Applied Health Research and Care) for Greater Manchester, which is a coordinated programme of research that aims to create, adapt and implement strategies to support socially disadvantaged people with long-term vascular conditions.

## Methods

The trial has received full ethical approval from the Health Research Authority (REC reference: 11/NW0855) and is being conducted in accordance with the UK Department of Health’s Research Governance Framework.

### Participants and recruitment

CKD is classified into five stages from stage 1 (mild) to stage 5 (established renal failure or end stage renal disease) based on estimated glomerular filtration rate (eGFR) [1,2,20]. Stages 3 to 5 may be defined by a reduction in eGFR alone, though for stages 1 and 2, other markers of kidney damage are required for a diagnosis of CKD [1,2,6]. Recognising evidence of increased risk of mortality with an eGFR <45 ml/min/1.73 m2 in all age groups, stage 3 CKD is split into two subcategories (3a and 3b) [1,2]. In addition, proteinuria (presence of excess protein in the urine) is also an independent predictor of mortality [21].

A total of 24 GP practices in the Greater Manchester area agreed to take part in an RCT evaluating a self-management intervention for vascular health in patients with stage 3 CKD [22]. Respecting GP judgement on eligibility, 440 out of 637 (69%) patients who agreed to be contacted were recruited between April and November 2012 with an average number of 16 patients per practice (range: 3–44). Four patients were excluded post-randomisation found to not meet the criteria of stage 3 CKD. Data on the total number of patients approached to take part was very patchy making it not possible to determine the overall response rate.

Thirteen GP practices had previously participated in an NIHR CLAHRC Renal Collaborative, which aimed to improve identification and management of CKD [23]. Inclusion criteria were a diagnosis of stage 3 CKD (stage 3a or 3b) as recorded on the general practice’s CKD register (a record of patients aged 18 years and over with stage 3–5 CKD which informs the UK’s pay-for-performance contract (QOF)). Efforts were made to ensure that patients had recently (ideally within the previous 6–8 weeks) attended their GP practice for a routine disease review appointment where their blood pressure had been taken and sign an informed consent form at the baseline assessment visit. Participants who were unable to communicate in English, lacked capacity to provide informed consent, or were in receipt of palliative care were excluded. Only one person per household was eligible to take part, to avoid potential contamination across trial arms. Detailed methods are reported in the trial protocol [22].

### Data collection

Baseline assessments were conducted within 6–8 weeks of a clinical appointment in primary care where baseline blood pressure was taken (Mean = 27.1 days prior to randomisation). Stage of CKD (3a and 3b) and evidence of proteinuria were also collected from general practice records. Patients completed a study questionnaire as part of the baseline assessment. This included the question ‘Please tell us if you have any of the following long-term medical conditions’, with a list of 17 long-term conditions for the patient to tick all that applied and free-text space for any additional long-term conditions (see Additional file 1). Self-report of CKD was defined by a ‘yes’ response to ‘Kidney Problems’. For consistency, if patients asked for further clarification about what was meant by ‘kidney problems’, interviewers were instructed to ask ‘Has your GP or other health professional told you that you have problems with your kidneys?’ Interviewers were instructed to offer no further prompts to patients. Other variables collected at baseline included self-reported demographics (age, gender, ethnicity, level of education completed and deprivation), and information on co-morbid (additional to CKD) long-term conditions and vascular risk factors, as well as measures of disease self-management ability (heiQ; [24]), health status (SF36; [25]) health related quality of life (EuroQol EQ-5D; [26]), anxiety (HADS-A; [27]), and health service utilisation. Further details of these measures are available in the trial protocol [22].

### Statistical analysis

Continuous variables were summarised using means and standard deviations (SDs), and categorical data with counts and percentages. Rates of missing data were low (<5% for all variables) and Expectation-Maximisation (EM) imputation was applied to impute missing values using the full set of baseline variables. Imputation and analyses were conducted using STATA IC (version 12.1) with an alpha level for significance of 5%. General practice was defined as a random effect in all the models and robust estimates of variance were used. The main trial was not powered for an analysis of CKD self-report predictors. However, post-hoc power analysis indicated that the sample of 436 with a 48% self-report rate, had 90% power to detect an odds-ratio of 1.38 between an explanatory factor and CKD self-report at alpha=5%. For a two-level predictor, this equates to an 8% difference between levels (i.e. 52% v 44%).

In order to investigate what factors were associated with self-reporting of CKD, univariate logistic regression with patients clustered by practice was used to explore the association between each explanatory variable and self-report of CKD. Multivariate logistic regression analysis was next applied using all variables from the univariate analyses with p values <=0.1 (to avoid prematurely excluding important associations) as predictors to identify independent predictors of self-report of CKD. A backwards elimination procedure was used to sequentially remove predictors with the highest p values until all remaining variables were significant at the p < 0.05 level. Predictor variables with more than two categories were modelled using sets of indicator variables. We used variance inflation factors (VIFs) to examine possible collinearity between explanatory variables.

To calculate overall predictive power for the logistic regression models we used the McKelvey and Zavoina R^2^, which estimates explained variance in a latent continuous variable underlying observed awareness (yes/no). We used this measure for ease of interpretation as it is comparable to explained variance in linear regression [28]. To assess if levels of CKD awareness differed between practices, we estimated the percentage of the total variance accounted for by differences between practices and compared this to zero using a likelihood-ratio chi-square test. We did this both with and without control for significant patient-level predictors of CKD self-report.

## Results

Baseline data are summarised in Table 1. A total of 436 eligible patients from 24 GP practices (70.6% of the 34 practices approached) provided baseline data. Practices had a mean list size of 5815 patients and we recruited an average of 16 patients per practice (range 3–44). 58.5% (n = 255) were female and patients were almost all of white ethnicity. The mean age of patients was 72.1 years. Patients reported a mean number of co-morbid long-term conditions (excluding kidney problems) of 3.5 (from the other 16 conditions listed and any additional long-term conditions reported in the free-text). 41.7% (n = 182) of patients had co-morbid established cardiovascular disease.

**Table 1:** **Baseline characteristics**

	**Total (n = 436)**	**Did not self-report kidney problems (n = 227)**	**Self-reported kidney problems (n = 209)**	**Odds-ratio (95% CI)**	**Univariate P-value**
Recruited from collaborative practice				1.18 (0.78, 1.80)	0.43
No	174 (39.9)	95 (54.6)	79 (45.4)		
Yes	262 (60.1)	132 (50.4)	130 (49.6)		
Gender				1.69 (1.14, 2.50)	0.009**
Male	181 (41.5)	108 (59.7)	73 (40.3)		
Female	255 (58.5)	119 (46.7)	136 (53.3)		
Age					0.24
<70	157 (36.0)	75 (47.8)	82 (52.2)	1.0	
70-79	178 (40.8)	95 (53.4)	83 (46.6)	0.80 (0.61, 1.05)	
80+	101 (23.2)	57 (56.4)	44 (43.6)	0.71 (0.39, 1.29)	
Ethnicity				5.54 (0.53, 57.81)	0.15
White	430 (98.6)	226 (52.6)	204 (47.4)		
Non-white	6 (1.4)	1 (16.7)	5 (83.3)		
Education				1.04 (0.70, 1.55)	0.85
No qualifications	192 (44.0)	101 (52.6)	91 (47.4)		
> = 1 qualification	244 (56.0)	126 (51.6)	118 (48.4)		
Index of multiple deprivation (IMD) (Higher score = greater deprivation)	16.95 (9.8, 31.7)	16.16 (9.6, 31.1)	18 (10.1, 31.7)	1.00 (0.99, 1.02)	0.55
Access internet for health information				1.23 (0.74, 2.05)	0.43
No	322 (73.9)	172 (53.4)	150 (46.6)		
Yes	114 (26.1)	55 (48.3)	59 (51.8)		
CKD stage				2.67 (1.96, 3.64)	<0.001***
3a	330 (75.7)	191 (57.9)	139 (42.1)		
3b	106(24.3)	36 (34.0)	70 (66.0)		
Proteinuria				1.95 (1.02, 3.69)	0.042*
No	388 (89.0)	209 (53.9)	179 (46.1)		
Yes	48 (11.0)	18 (37.5)	30 (62.5)		
Co-morbid long-term conditions					0.020*
<=2	136 (31.2)	79 (58.1)	57 (41.9)	1.0	
3 or 4	192 (44.0)	100 (52.1)	92 (47.9)	1.28 (0.84, 1.94)	
5+	108 (24.8)	48 (44.4)	60 (55.6)	1.73 (1.17, 2.57)	
Co-morbid established cardiovascular disease				1.11 (0.89, 1.39)	0.36
No	254 (58.3)	135 (53.2)	119 (46.9)		
Yes	182 (41.7)	92 (50.6)	90 (49.5)		
High blood pressure				0.91 (0.62, 1.33)	0.61
No	174 (39.9)	88 (50.6)	86 (49.4)		
Yes	262 (60.1)	139 (53.1)	123 (47.0)		
Prostate and urological problems				1.15 (0.61, 2.18)	0.67
No	387 (88.8)	203 (52.5)	184 (47.6)		
Yes	49 (11.2)	24 (49.0)	25 (51.0)		
Diabetes				0.84 (0.55, 1.28)	0.41
No	335 (76.8)	171 (51.0)	164 (49.0)		
Yes	101 (23.2)	56 (55.5)	45 (44.6)		
Arthritis or painful joints, back trouble, osteoporosis				1.38 (0.82, 2.33)	0.23
No	150 (34.4)	86 (57.3)	64 (42.7)		
Yes	286 (65.6)	141 (49.3)	145 (50.7)		
≥1 Other (COPD, stomach ulcer, reflux or IBS, CFS, neurological condition, thyroid problems, skin problems, cancer, haematology, ENT, eye problems or dementia).				1.34 (0.95, 1.90)	0.094
No	166 (38.1)	94 (56.6)	72 (43.4)		
Yes	270 (61.9)	133 (49.3)	137 (50.7)		
General health (Higher score = better general health)	2.75 ± 0.94	2.89 ± 0.96	2.60 ± 0.90	0.72 (0.60, 0.85)	<0.001***
Energy & Vitality (Higher score = greater energy levels)	50.78 ± 22.83	55.39 ± 21.65	45.77 ± 23.07	0.98 (0.97, 0.99)	<0.001***
HADS –anxiety					<0.001***
0-3	183 (42.0)	107 (58.5)	76 (41.5)	1	
4-7	151 (34.6)	86 (57.0)	65 (43.1)	1.06 (0.61, 1.86)	
8+	102 (23.4)	34 (33.3)	68 (66.7)	2.82 (1.46, 5.43)	
SCDSC self-care (Higher score = greater self-care skills)	4.35 ± 1.23	4.40 ± 1.24	4.29 ± 1.21	0.93 (0.85, 1.02)	0.13
> 1 cardiovascular risk factor (Diabetes, hypertension or smoking)				1.18 (0.78, 1.77)	0.44
No	352 (80.7)	186 (52.8)	166 (47.2)		
Yes	84 (19.3)	41 (48.8)	43 (51.2)		
HeiQ (Self-monitoring and insight) (Higher score = greater self-monitoring)	68.97 ± 12.26	70.74 ± 11.61	67.05 ± 12.68	0.97 (0.96, 0.99)	0.002**
HeiQ (Health services navigation) (Higher score = greater health service navigation)	69.67 ± 15.29	71.31 ± 15.05	67.9 ± 15.39	0.99 (0.97, 1.00)	0.01*
GP contact in previous 6 months	2 (1, 4)	2 (1, 3)	2 (1, 4)	1.07 (1.01, 1.14)	0.027*
Nurse visits in previous 6 months	2 (1, 3)	2 (1, 3)	2 (1, 3)	1.03 (0.98, 1.09)	0.29
Total hospital visits in previous 6 months	1 (0, 2)	0 (0, 2)	1 (0, 2)	1.03 (0.99, 1.06)	0.12

Data are mean ± SD, number (%) or median (25^th^, 75^th^ percentile) when data are skewed. Patients aware of kidney problems versus those not aware. *p < 0.05, **p < 0.01, ***p < 0.001.

Just over half of patients (52.1%, n = 227) did not self-report CKD. Rates of self-report varied between practices, from a low of 22.7% (5 out of 22 patients) to a high of 75% (3 out of 4 patients).

In univariate analysis, patients who self-reported CKD were significantly (p < 0.05) more likely to be female, have CKD stage 3b, have proteinuria, poorer self-reported general health, lower energy and vitality, higher levels of general anxiety, lower levels of self-monitoring and insight (heiQ; [24]), poorer health services navigation skills (heiQ; [24]), and more frequent contact with their GP (see OR’s in Table 1). Patients with greater numbers of long-term conditions were also more likely to self-report CKD. However, age, socioeconomic status, cardiovascular risk factors and established cardiovascular disease, did not show significant relationships with self-report of CKD (p > 0.05). There was no significant difference in self-report of CKD between patients recruited from CLAHRC collaborative and non-collaborative practices [23].

**Figure 1:**
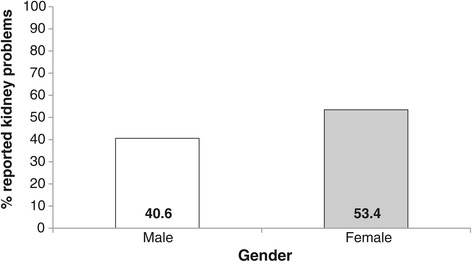
**Bar graph indicating the percentage** ^**1**^ **of patients reporting kidney problems by gender.** ^1^Percentages controlled for other significant predictors (CKD stage and HADS-anxiety).

**Figure 2:**
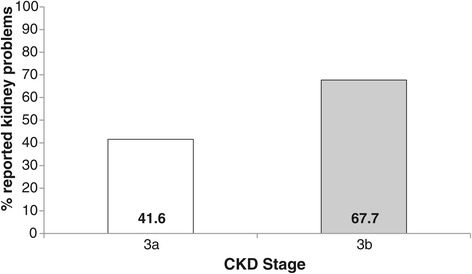
**Bar graph indicating the percentage** ^**1**^ **of patients reporting kidney problems by CKD stage.** ^1^Percentages controlled for other significant predictors (gender and HADS-anxiety).

**Figure 3:**
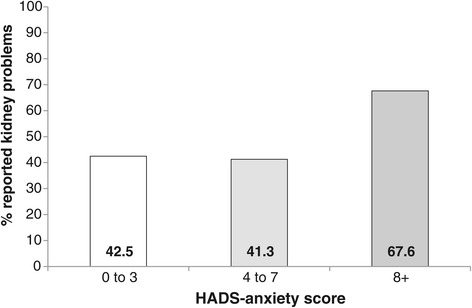
**Bar graph indicating the percentage** ^**1**^ **of patients reporting kidney problems by HADS-Anxiety score.** ^1^Percentages controlled for other significant predictors (gender and CKD stage).

Variance inflation factors amongst the explanatory variables entered into the multivariate analysis were all low (maximum =2.3), indicating acceptable multicollinearity [29]. Since both stage of CKD and proteinuria were potential explanatory variables, we also added their interaction term to the initial model. In multivariate analysis, just three variables remained in the model as independent predictors of self-report of CKD: stage of CKD, gender and anxiety. Women were more likely to self-report CKD than men (OR = 1.68, 95% CI 1.18-2.39). The relationship with anxiety was nonlinear: patients with low levels of anxiety (HADS <=3) and moderate anxiety scores (HADS =4 to 7) had very similar degrees of awareness, but patients with clinical levels of anxiety (HADS >=8) were much more likely to show awareness (OR = 2.83, 95% CI 1.44-5.54) compared to low anxiety. The association with stage of CKD indicated considerably higher levels of self-report amongst patients at stage 3b compared to stage 3a (OR = 2.94, 95% CI 2.16-4.01) (see Table 2). Subgroup differences are illustrated in Figures 1, 2 and 3. The model as a whole explained approximately 13% of the variance in patient awareness (McKelvey and Zavoina’s R^2^ = .13).

**Table 2:** **Multivariate analysis of awareness of kidney problems**

**Variable**	**Odds-ratio (95% CI)**	**P-value**
Gender - Female	1.68 (1.18, 2.39)	0.004**
HADS – Anxiety		<0.001***
0- 3	1	
4-7	0.95 (0.56,1.61)	
8+	2.83 (1.44, 5.54)	
CKD Stage 3b	2.94 (2.16, 4.01)	<0.001***

**p < 0.01, ***p < 0.001.

Both with and without control for these patient factors, the percentage of total variance accounted for by differences in levels of CKD awareness between practices was very low, less than 1%, and not statistically significant (p = 0.49 and p = 0.41, respectively).

## Discussion

An analysis of baseline data from 436 patients with a diagnosis of CKD stage 3 from an RCT [22] was conducted. Patients were elderly (64% of patients were aged over 70 years) and had relatively high numbers of additional long-term conditions. Despite being recruited from a CKD register, around half of all patients did not self-report CKD.

Self-report of CKD did not appreciably differ between individual practices, or between CLAHRC Renal Collaborative practices and non-collaborative practices, suggesting that patient level characteristics may be more important in predicting self-report of CKD than practice level differences. Patients who were female and those with CKD stage 3b, were significantly more likely to self-report CKD. Using a criteria of a HADS anxiety score of 8 or more to indicate anxiety above normal levels [30,31], the large majority of patients (n = 334, 76.6%) fell within a normal range of anxiety symptoms. However, despite this, patients with above normal HADS anxiety scores were significantly more likely to self-report CKD.

### Comparison with existing literature

Our findings are consistent with previous research which has shown that awareness of early stage CKD is variable [8,16], clinical discussion about CKD infrequent [9], and use of the QOF registers for CKD inconsistent [6]. The findings may reflect wider issues around the merits of discussing early stage CKD with patients who are often elderly and multimorbid, with concerns of unnecessary ‘disease labelling’ [2,16,17,19,32]. Nevertheless, evidence suggests that patients, regardless of age, expect to be informed about diagnostic information [33] and our findings suggest that procedures to meet this expectation may be variable and inconsistent.

Patients with stage 3b CKD were significantly more likely to self-report CKD than stage 3a patients which is consistent with evidence of controversy about the benefits of discussing CKD with patients, particularly those who have stage 3a CKD [15,19,32]. It may be that conversations about CKD with stage 3a patients are framed as reassurance [19] and thus provide ambiguous understanding and inconsistent knowledge of the relevance of CKD to patients overall health. Recognising the importance of monitoring for disease progression, future research would benefit from identifying whether patients with declining (versus stable) renal function are more or less likely to self-report CKD.

Previous research suggests that younger, male patients are more likely to be aware of CKD than older female patients [8,16]. However, a substantially higher percentage of females in our sample self-reported CKD compared to males, and we did not find age a significant predictor of self-report. Our elderly sample included few patients under 65 years, which may help explain why we did not find that self-report of CKD decreased significantly with increasing age as expected.

In contrast to previous US-based studies [7,16] we did not find established vascular disease or risk factors including diabetes to be significantly associated with self-reporting of CKD. Our results were similar to a recent UK-based study [8] and may suggest that conversations with health-care professionals around vascular health could be seen as somewhat separate from conversations around CKD.

### Strengths and limitations

The inclusion criteria (patient on a CKD register and attendance at a recent disease review appointment) ensured that all patients in our sample were diagnosed with stage 3 CKD and were being actively managed in primary care.

However, the use of baseline data from a trial leads to certain limitations. Samples participating in trials in primary care are generally atypical. Over two thirds of eligible patients participated, but we were unable to assess non-response as accurate data on the total number of patients approached were not available, and we cannot make strong statements about awareness in the wider population based on such a sample. However, the focus of the study was on *associations* between patient and practice characteristics and self-report of CKD. Such associations are less likely to be affected by low proportions of patients taking part in a trial, although appropriate caution must be exercised in making generalisations.

The validity of the analyses is dependent on the validity of the measures used. We recognise that patient self-reporting of CKD in terms of ‘kidney problems’ does not necessarily equate with lack of clinical dialogue. Previous research has highlighted that discussions around CKD by GPs and practice nurses with patients are often normalised and framed in the form of reassurance [19], for example by framing deterioration of kidney function as a normal consequence of ageing, thereby perhaps decreasing the likelihood that patients self-report CKD. Observational analysis is needed to illuminate the framing of CKD during clinical encounters [34]. Nevertheless, our results are comparable to a previous study by McIntyre *et al.* [8] who utilised a similar self-report measure and found that 41% of patients with stage 3 CKD were unaware of their diagnosis. Further research is required to capture more detailed accounts of patients knowledge not only of CKD but also awareness around self-management.

Due to the cross-sectional nature of this study, we have to be cautious when interpreting the direction of the relationship between anxiety levels and self-report of CKD. Although the majority of patients were within the normal range of anxiety symptoms, those who reported higher levels of anxiety were more likely to be aware of kidney problems. Three potential explanations for why anxiety may be significantly higher in patients that self-report CKD include, a) receiving a CKD diagnosis increases anxiety, b) anxiety promotes information-seeking and active communication with a healthcare professional about CKD, or c) anxiety inflates the likelihood that patients self-report conditions in general, including CKD. However, not all patients with higher levels of anxiety reported kidney problems. This might be indicative of a reassuring conversation around kidney health due to healthcare professionals sensitivity to the patient’s anxiety. Further research will be required in order to clarify these relationships.

The BRIGHT trial recruited patients with a diagnosis of stage 3 CKD but did not collect data on evidence of a rate of decline in renal function (eGFR of >5 ml/min/1.73 m2 within 1 year, or >10 ml/min/1.73 m2 within 5 years) [1]. Although data was collected on staging (3a or 3b) and evidence of proteinuria, we acknowledge that the study was unable determine whether patients with evidence of disease progression are more or less likely to self-report CKD.

### Implications

Despite efforts to improve the identification of stage 3 CKD in primary care, over half of the patients recruited from a CKD register as part of a wider RCT, did not self-report CKD. The low levels of reporting of CKD raises issues around the optimisation of interventions and trial designs in this population [35]. Although efforts were made to build on existing clinical practice, these findings from baseline analysis, irrespective of final trial outcomes, suggest that information resources to support understanding of CKD and the maintenance of vascular health may benefit from closer alignment with existing clinical encounters.

Our data suggests the need for greater consistency in discussions around kidney health. Broadening and tailoring the scope of a CKD diagnosis by framing it in the context of patient’s overall health may assist practitioners in ensuring meaningful and relevant conversations about kidney health, which are aligned with existing clinical encounters such as cardiovascular disease reviews, medication reviews and prevention of acute kidney injury (AKI) during phases of acute illness (e.g. sepsis due to flu). Recently published guidelines on the prevention, detection and management of AKI may support this shift in clinical dialogue and assist with shared decision making [36]. As recommended by NICE, patient involvement in research is required in order to support the development and communication of more personalised information around the management of CKD [1].

## Conclusions

Our data indicates that patient self-report of CKD is variable, suggesting a need for greater consistency in discussions around kidney health. In light of our findings and recent research and commentary highlighting a need for effective strategies to improve the management of CKD and prevention of AKI [8,32,37-39], we argue that information exchange concerning early stage CKD may be improved by broadening the reasons for communication. Further patient-orientated research is needed to support the development and tailoring of information to ensure maintenance of kidney health, whilst minimising health burden and anxiety.

## Additional file

Additional file 1:
**Complete list of the seventeen long-term conditions included as self-report options for participants in the baseline questionnaire.**
