# Supplementary material for: Predictors of patient self-report of chronic kidney disease: baseline analysis of a randomised controlled trial
Source: BMC Fam Pract. 2014 Nov 30;15:196. doi: 10.1186/s12875-014-0196-3 (PMC4260196; doi:10.1186/s12875-014-0196-3)
Supplement: Additional file 1: — Complete list of the seventeen long-term conditions included as self-report options for participants in the baseline questionnaire. [file 12875_2014_196_MOESM1_ESM.docx]

| “Please tell us if you have any of the following long-term medical conditions” | |
| --- | --- |
| 1 | Angina or heart attack |
| 2 | Irregular Heartbeat (such as AF) |
| 3 | Heart Failure |
| 4 | Stroke, TIA's (Transient Ischaemic Attacks) or mini strokes |
| 5 | Peripheral vascular (arterial) disease or 'narrowing of the arteries of the legs' |
| 6 | High blood pressure |
| 7 | Kidney problems |
| 8 | Prostate or urological problems |
| 9 | Diabetes |
| 10 | Breathing problems such as COPD, asthma or emphysema |
| 11 | Stomach or peptic ulcer, reflux, IBS or other abdominal (tummy) problems |
| 12 | Arthritis or painful joints, back trouble, osteoporosis |
| 13 | Chronic fatigue syndrome, ME or fibromyalgia |
| 14 | Anxiety, depression or stress |
| 15 | Neurological conditions such as Multiple Sclerosis or Parkinson's disease |
| 16 | Thyroid problems |
| 17 | Skin problems (e.g. eczema or psoriasis) |
| A further five long-term condition categories were computed following inclusion of frequent free-text long-term conditions. These included: | |
| 18 | Cancer |
| 19 | Haematology |
| 20 | Ear, nose and throat |
| 21 | Eye problems |
| 22 | Dementia |
| Further long-term conditions reported in the free-text and not already counted within the existing or additional categories were also included in the ‘co-morbid long-term conditions’ count. | |
